# Thermogenic effect of an acute ingestion of a weight loss supplement

**DOI:** 10.1186/1550-2783-6-1

**Published:** 2009-01-06

**Authors:** Jay R Hoffman, Jie Kang, Nicholas A Ratamess, Stefanie L Rashti, Christopher P Tranchina, Avery D Faigenbaum

**Affiliations:** 1 Department of Health and Exercise Science, The College of New Jersey, PO Box 7718, Ewing, New Jersey 08628, USA

## Abstract

**Background:**

The purpose of this study was to examine the acute effects of a weight loss supplement on resting oxygen uptake (VO_2_), respiratory quotient (RQ), caloric expenditure (kcal), heart rate (HR), and blood pressure (BP) in healthy and physically active individuals.

**Methods:**

Ten subjects (5 male, 5 female; 20.2 ± 1.2 y; 172.2 ± 8.9 cm; 71.5 ± 17.2 kg; 17.3 ± 2.6% body fat) underwent two testing sessions administered in a randomized and double-blind fashion. During each session, subjects reported to the Human Performance Laboratory after at least 3-h post-absorptive state and were provided either 3 capsules of the weight loss supplement (SUP), commercially marketed as Meltdown^® ^or 3 capsules of a placebo (P). Subjects then rested in a semi-recumbent position for three hours. VO_2 _and HR were determined every 5 min during the first 30 min and every 10 min during the next 150 min. BP was determined every 15 min during the first 30 min and every 30 min thereafter. The profile of mood states was assessed every 30 min.

**Results:**

Area under the curve analysis revealed a significant 28.9% difference in VO_2 _between SUP and P for the three hour study period. In addition, a significant difference in energy expenditure was also seen between SUP (1.28 ± 0.33 kcal·min^-1^) and P (1.00 ± 0.32 kcal·min^-1^). A trend (p = 0.06) towards a greater utilization of stored fat as an energy source was also demonstrated (0.78 ± 0.23 kcal·min^-1 ^and 0.50 ± 0.38 kcal·min^-1 ^in P and SUP, respectively). Significant elevations in HR were seen during hours two and three of the study, and significantly higher average systolic BP was observed between SUP (118.0 ± 7.3 mmHg) and P (111.4 ± 8.2 mmHg). No significant differences were seen in diastolic blood pressure at any time point. Significant increases in tension and confusion were seen in SUP.

**Conclusion:**

Results indicate a significant increase in energy expenditure in young, healthy individuals following an acute ingestion of a weight loss supplement. In addition, ingestion of this supplement appears modify mood and elevate HR and systolic BP following ingestion.

## Background

The use of energy drinks and capsules have recently been shown to be the most popular supplement besides multivitamins in the American adolescent and young adult populations, as more than 30% of American adolescents self-admit to using thermogenic supplements on a regular basis [1]. The primary reason for use of these supplements is thought to be related to their desire to reduce or control body fat [1-3]. A number of herbal ingredients have been proposed as being effective agents in increasing energy expenditure and reducing body fat [4]. Although studies examining the thermogenic effect (i.e. increase in caloric expenditure) from high-energy supplements are limited, several recent investigations have suggested that the combination of thermogenic agents in a supplement may be more effective in increasing the thermogenic effect than a single herbal ingredient [5,6].

Caffeine has been shown to be an effective supplement in enhancing lipolysis, fat oxidation, and reducing glycogen breakdown [7,8], however when combined with other thermogenic agents its effectiveness appears to be magnified [5,6]. For many years caffeine was often combined with ephedra that resulted in an enhanced metabolic response leading to greater body fat loss [9,10]. However, as a result of the Federal Drug Administration's ban on ephedrine alkaloids in 2004 the use of alternative therapeutic means to combat obesity has been examined. Synephrine is a mild stimulant and is thought to contribute to appetite suppression, increased metabolic rate and lipolysis [11]. Synephrine is thought to stimulate specific adrenergic receptors (β-3) that stimulate fat metabolism without any of the negative side effects (i.e., elevated systolic blood pressure, heart rate and thermogenic strain) generally associated with compounds that stimulate the other adrenergic receptors [12]. Recent research has suggested that to maximize the effectiveness of synephrine as an effective weight loss supplement it may need to be combined with other herbal products [13]. Some of these products may include yohimbine, yerba mate extract, hordenine and methyl tetradecylthioacetic acid. All of which have been shown to play a role in enhancing lipolysis and increasing energy expenditure [14-16].

In addition to increasing thermogenesis many of these supplements may also contain herbal ingredients whose primary role is to enhance mood. Phenylethylamine is an example of an endogenous neuroamine that has been included in weight loss supplements. Several studies have shown that phenylethylamine can relieve depression and improve in clinical populations [17,18]. Much of the work on phenylethylamine was based upon research that demonstrated that a deficit in phenylethylamine can be responsible for depression [19]. As a result, it has been used both clinically to treat depression, and in energy supplements to enhance mood.

Considering the high propensity in the use of these thermogenic supplements, research is warranted concerning the efficacy of these energy supplements. Thus, the purpose of this study was to examine the acute effect of a weight loss supplement containing several herbal and botanical ingredients on resting oxygen uptake, respiratory quotient, caloric expenditure, heart rate, and blood pressure in healthy and physically active individuals.

## Methods

### Subjects

Ten subjects (5 male, 5 female; 20.2 ± 1.2 y; 172.2 ± 8.9 cm; 71.5 ± 17.2 kg; 17.3 ± 2.6% body fat) underwent two testing sessions administered in a randomized and double-blind fashion. Following an explanation of all procedures, risks, and benefits associated with the experimental protocol, each subject gave his or her written informed consent to participate in this study. The Institutional Review Board of The College of New Jersey approved the research protocol. Subjects who were pregnant, smokers or taking regular medication except birth control pills were excluded from the study. Subjects with any known metabolic or cardiovascular disease, or psychiatric disorder were also excluded. Subjects were also required to have been free of any nutritional supplements or ergogenic aids for 6 weeks preceding the study, and were asked to refrain from taking any additional supplement during the duration of the study.

### Study design

The study followed a double-blind, crossover design. Subjects reported to the Human Performance Laboratory on two separate days. Each testing session was separated by an average of 3 days (3.4 ± 2.0 d). Subjects were instructed to refrain from consuming any caffeine products on the day of each testing session and from performing any strenuous physical activity for the previous 12 hours. In addition, subjects were instructed to be at least 3 hours post-absorptive state prior to each trial. Following a 30 min rest period subjects were randomly provided either the supplement (SUP) or the placebo (P). During the second visit to the laboratory subjects were provided with the opposite treatment.

### Metabolic measures

Immediately following supplement ingestion subjects were fitted with a Medgraphics preVent™ pneumotach (Medical Graphics Corporation, St. Paul, MN) to measure oxygen consumption (VO_2_) and respiratory quotient (RQ) through open-circuit spirometry using a metabolic measurement cart (CPX Ultima™ series, Medical Graphics Corporation, St. Paul, MN) with breath by breath analysis. Machine calibration was performed prior to each session. Measures of VO_2_, RQ, energy expenditure, fat oxidation rate and heart rate (HR) using a wireless HR monitor (Pacer, Polar CIC, Inc., Port Washington, NY) were obtained one minute following supplement or placebo consumption, every 5 min for the first 30 min, and every 10 min thereafter until 180 min post consumption. Blood pressure (BP) was measured using a sphygmomanometer and ausculatory method at 15 min and 30 min post ingestion, and then for every 30 min until data collection concluded.

### Questionnaires

The profile of mood states (POMS) was administered seven times during each testing session. The initial POMS administration was given as the subject reported to the Human Performance Laboratory, and every half hour for the three hour period following supplement ingestion. All questionnaires were performed under controlled conditions (a quiet room alone with the investigator) and the same researcher performed all test administrations.

The POMS consists of 65 words or phrases in a Likert format questionnaire which provides measures of specific mood states. It provides measures of tension, depression, anger, vigor, fatigue and confusion. A total mood score is computed by subtracting vigor from the sum of the five other negative measures and adding 100 to avoid a negative result. McNair et al., [20] has reported internal consistency of measures ranging between 0.85 to 0.95 and test-retest reliability estimates ranging between 0.65 to 0.74. These lower coefficients of stability are thought to be indicative of transient and fluctuating characteristics of mood states. During all test administrations participants were asked to describe their feelings upon how they were feeling at that moment.

### Supplement

On each visit subjects ingested either 3 capsules of Meltdown^® ^(SUP) or a placebo (PL). Meltdown^® ^contains the following: 317 mg of a proprietary blend of caffeine anhydrous, α-methyl tetradecylthioacetic acid, yerba mate extract, and cAMP; 20 mg of methyl-synephrine HCl, 138 mg of a proprietary blend of β-methylphenylethylamine and methyl-β-phenylethylamine; 9 mg of a proprietary blend of 11-hydroxy yohimbine, yohimbine HCl, and α-yohimbine; 20 mg of methyl-hordenine HCl. The placebo was similar in appearance and texture to Meltdown^®^, but contained only an inert substance.

### Statistical analyses

Statistical analysis of the data was accomplished using a repeated measures analysis of variance. In the event of a significant F-ratio, LSD post-hoc tests were used for pairwise comparisons. RQ, HR, BP and POMS were averaged over each hour and for the entire 3-hour study period. Comparisons of the average 3-hour measures were analyzed using dependent T-tests. A criterion alpha level of p ≤ 0.05 was used to determine statistical significance. All data are reported as mean ± SD.

## Results

**Figure 1:**
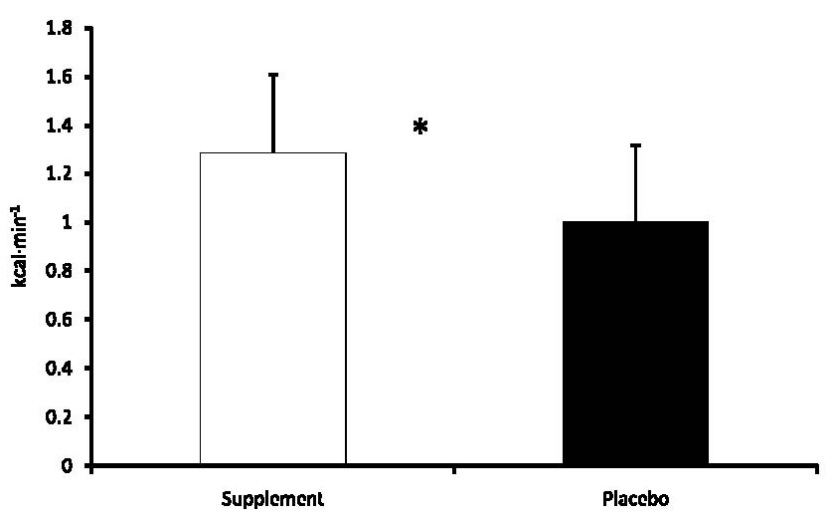
**Average 3-Hour Energy Expenditure**. * = Supplement significantly (p < 0.05) different than Placebo: Data are reported mean ± SD.

A significant difference (p = 0.01) in average energy expenditure was seen between SUP (1.28 ± 0.33 kcal·min^-1^) and P (1.00 ± 0.32 kcal·min^-1^) during the entire 3-hr period (see Figure 1). Significant differences in the average energy expenditure between the groups were also seen at each hour of the protocol (see Table 1).

**Table 1:** Average hourly cardiovascular and energy expenditure measures

Variable		Baseline	Hour 1	Hour 2	Hour 3
Heart Rate (b·min^-1^)	SUP	70.4 ± 9.4	71.2 ± 11.2	74.3 ± 12.6 *	72.3 ± 9.1*
	
	P	70.0 ± 6.2	67.9 ± 7.1	65.3 ± 5.7	64.8 ± 5.8

Systolic Blood Pressure (mmHg)	SUP	112.7 ± 9.9	115.8 ± 7.7 *	121.2 ± 6.8 *	119.3 ± 8.9 *
	
	P	110.8 ± 9.6	111.7 ± 9.0	109.7 ± 7.3	111.7 ± 7.9

Diastolic Blood Pressure (mmHg)	SUP	74.0 ± 6.0	76.7 ± 9.1	76.1 ± 7.5	76.3 ± 7.7
	
	P	75.4 ± 7.5	76.1 ± 9.6	75.7 ± 5.9	74.9 ± 6.9

Energy Expenditure (kcal·min^-1^)	SUP	1.16 ± .36	1.25 ± .39 *	1.29 ± .34 *	1.31 ± .28 *
	
	P	1.00 ± .35	0.96 ± .27	1.03 ± .35	1.05 ± .37

RQ	SUP	0.89 ± .09	0.86 ± .05	0.80 ± .04 *	0.79 ± .04 *
	
	P	0.89 ± .07	0.87 ± .09	0.87 ± .07	0.86 ± .07

*P < 0.05, SUP > P; SUP = Supplement; P = Placebo

**Figure 2:**
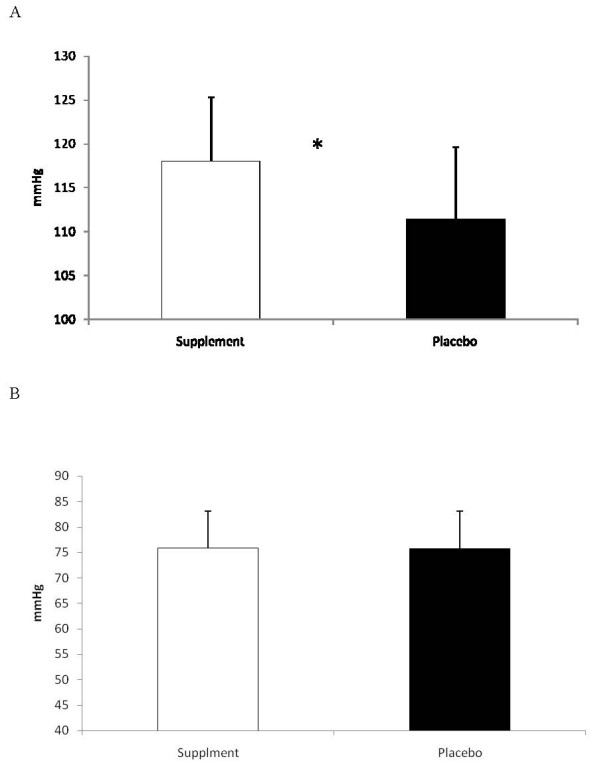
**a: Average 3-Hour Systolic Blood Pressure**. * = Supplement significantly (p < 0.05) different than Placebo: **2b**: Average 3-Hour Diastolic Blood Pressure. Data are reported mean ± SD.

The average hourly cardiovascular response to the study protocol is seen in Table 1. Heart rate was significantly higher during hours two and three for SUP compared to P. The average systolic blood pressure response in SUP was significantly higher at each hour compared to P. The average systolic blood pressure response for the 3-hr protocol was also significantly greater (p = 0.002) for SUP than P (see Figure 2a). No difference between the groups was seen in the diastolic blood pressure response (Table 1 and Figure 2b).

**Figure 3:**
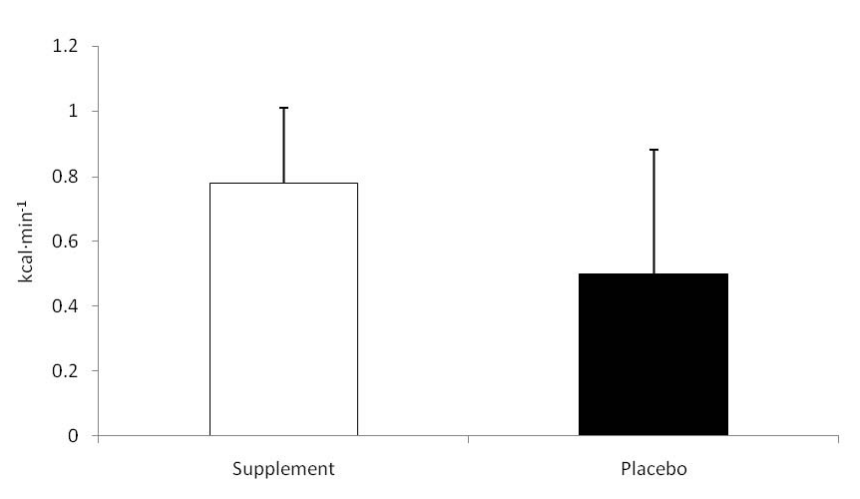
**Average 3-Hour Fat Utilization**. Data are reported mean ± SD.

The average RQ was significantly lower for SUP than P at hours two and three (see Table 1). In addition, a trend (p = 0.06) towards a greater utilization of stored fat as an energy source, expressed as energy expenditure from fat, was also demonstrated during the 3-hr study protocol for SUP compared to P (see Figure 3).

**Figure 4:**
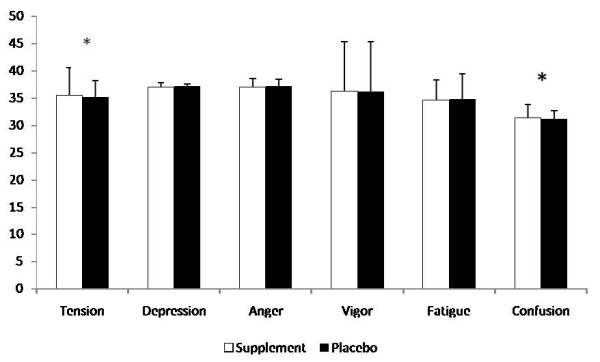
**Average Profile of Mood States**. * = Supplement significantly (p < 0.05) different than Placebo. Data are reported mean ± SD.

Comparisons between groups in the average profile of mood states scores can be observed in Figure 4. No significant differences were seen in the average score for the mood states depression, anger, vigor, and fatigue. However, a significantly higher average tension and confusion score was observed during SUP compared to P.

## Discussion

The results of this study indicate that a weight loss supplement containing anhydrous caffeine, synephrine, tetradecylthioacetic acid, yerba mate extract, methylphenylethylamine, yohimbine, and hordenine is effective in increasing acute energy expenditure in young, healthy individuals. Ingestion of this supplement also resulted in significant elevations in heart rate and systolic blood pressure indicating a strong inotropic response to this supplement. In addition, acute ingestion of this supplement increased tension and confusion among subjects.

Comparisons between weight loss supplements need to be performed carefully considering the large variability seen in ingredients or different concentrations of ingredients. Previous research has shown that acute ingestion of caffeine, synephrine and other herbal ingredients in a coffee supplement significantly increased energy expenditure 12% among healthy, lean college students [6]. In this present study, using a similar subject population the combination of anhydrous caffeine, synephrine and different herbal ingredients resulted in a 29% increase in energy expenditure. Although differences could be related to differences in concentrations of similar ingredients within each supplement, the difference between these studies is likely related to the differences in co-ingredients within the supplement.

Caffeine and herbal supplements have been shown to increase resting metabolic rate for up to three hours following ingestion [6,10,21]. These effects have been shown in supplements combining caffeine with ephedra and black tea [10,21], and with caffeine combined with synephrine, garcinia cambogia and chromium polynicotinate [6]. The greater energy expenditure seen in this study compared to others is likely related to the additional ingredients in this supplement that have previously been found to enhance metabolism and therefore have a synergistic effect with known thermogenic ingredients such as caffeine.

Synephrine is a mild stimulant that comes from the fruit *citrus aurantium *(bitter orange). It is the predominant alkaloid from this fruit, and is thought to stimulate fat metabolism [12]. Hordenine is an alkaloid that occurs naturally in grains, sprouting barley and certain grasses, but it is also found in small quantities in citrus aurantium [22]. When infused to horses it has been shown to increase respiratory and heart rates, however when provided orally no significant cardiorespiratory changes were seen [23]. Other studies have suggested that hordinine can exert its stimulating effect by inhibiting norepinephrine uptake [15]. Although human studies with hordinine are very limited, it is likely it works synergistically with synephrine to enhance the sympathetic response.

The elevated blood pressure and heart rate responses seen during the SUP treatment are similar to other studies examining weight loss supplements containing adrenergic amines [5,6,13,24]. Synephrine is thought to increase lipolysis and minimize the cardiovascular effect typical of adrenergic amines [12], however synephrine has also been shown to stimulate peripheral α-1 receptors resulting in vasoconstriction and elevations in blood pressure [25]. Although synephrine ingested alone may not alter blood pressure, when ingested in combination with other active herbal ingredients does appear to elevate blood pressure and heart rate [5,6]. The combination of synephrine, caffeine and hordinine used in this study appears to cause significant elevations in the cardiovascular response to supplement ingestion.

The trend towards a greater utilization of stored fat as the primary energy source, as evidenced by a lower RQ, in this study is likely related to the combination of yohimbine, yerba mate extract and tetradecylthioacetic acid. The precise concentrations of yohimbine and its metabolites in this supplement are not known, thus discussion concerning how these differences may have effected changes in fat mobilization would only be speculative. Yohimbine is a selective α-adrenoceptor antagonist that has been shown to be effective in enhancing lipid metabolism [16,26]. However, the extent of yohimbine's effect may have been modulated by its various metabolites within the supplement. No differences in RQ between the groups were seen in the first hour following supplementation but significant differences were seen at hours two and three. This may be reflective of differences in α-2 adrenoceptor blocking potency and half-life between the metabolites of yohimbine [27]. Although yohimbine is a more potent α-adrenoceptor antagonist than its metabolites, it is metabolized more quickly.

Yerba mate extract made from the leaves of the tree Ilex paraguariensis has been shown to suppress appetite and prevent diet-induced obesity in rats [28] and humans [14]. It is thought to cause weight reduction by delaying gastric emptying [14] and its effects may be enhanced by caffeine [4]. Although it is proposed to have several potential health benefits besides weight loss [29], its role in elevating energy expenditure or increasing lipolysis is not well understood, and may be negligible. Tetradecylthioacetic acid has been shown to be effective in enhancing fatty acid metabolism [30].

The addition of phenylethylamine as an ingredient was thought to enhance the mood of subjects using this supplement. Phenylethylamine has been shown to produce relief of depression among a clinical population, even in those that were unresponsive to standard treatments [18]. An advantage in the use of phenylethylamine is thought to be related to the beneficial mood improvements seen without producing a tolerance often associated with amphetamines [18]. The mechanism of its effect appears to be related to the stimulation of dopamine release [31]. This may contribute to an improved mood state and has also been shown to potentially reduce appetite [32]. In addition, phenylethylamine may also stimulate lipolysis through its ability to stimulate catecholamine release and delay reuptake [33]. The results of this study indicate that phenylethylamine did not affect mood, but may have contributed to the greater reliance on fat as an energy source. Considering the various ingredients within this supplement, it is possible that the greater tension and confusion seen in SUP may have been a result of the adrenergic stimulants contained in the supplement.

In conclusion the results of this study indicate that following an acute ingestion of a weight loss supplement containing anhydrous caffeine, synephrine, tetradecylthioacetic acid, yerba mate extract, methylphenylethylamine, yohimbine, and hordenine leads to a significant increase in energy expenditure in young, healthy individuals. In addition, ingestion of this supplement stimulates elevations in heart rate and blood pressure for three hours, while increasing feelings of tension and confusion. Individuals who have been diagnosed with cardiovascular disease need to be aware of the significant cardiovascular effects resulting from use of this supplement. Additional research is warranted concerning the long-term effects of consumption of this supplement, and whether such supplementation can translate into weight loss or improved body composition.

## Competing interests

Vital Pharmaceuticals. (Davie, FL) provided funding for this project. All researchers involved independently collected, analyzed, and interpreted the results from this study and have no financial interests concerning the outcome of this investigation. Publication of these findings should not be viewed as endorsement by the investigator, The College of New Jersey or the editorial board of the Journal of International Society of Sports Nutrition.

## Authors' contributions

JRH was the primary investigator, obtained grant funds for project, designed study, supervised all study recruitment, data/specimen analysis, statistical analysis and manuscript preparation. JK, NAR, and ADF were co-authors, oversaw all aspects of study including recruitment, data/specimen analysis, and manuscript preparation. SCR, and CPT were co-authors, assisting with data collection and data analysis.
